# Changes in the quality of oil extracted by hot pressing from black cumin (*Nigella sativa*) seeds and by solvent from the obtained cake during refining

**DOI:** 10.1002/fsn3.4025

**Published:** 2024-02-20

**Authors:** Solmaz Abedinzadeh, Mohammadali Torbati, Sodeif Azadmard‐Damirchi, Fataneh Hashempour‐Baltork

**Affiliations:** ^1^ Department of Food Science and Technology, Faculty of Nutrition and Food Science Tabriz University of Medical Sciences Tabriz Iran; ^2^ Student Research Committee Tabriz University of Medical Sciences Tabriz Iran; ^3^ Department of Food Science and Technology, Faculty of Agriculture University of Tabriz Tabriz Iran; ^4^ Halal Research Centre of IRI, Iran Food and Drug Administration Ministry of Health and Medical Education Tehran Iran

**Keywords:** oil extraction, press, quality, refining, stability

## Abstract

In this study, oil was extracted from black cumin (*Nigella sativa*) seed (BCS) by press, and oil was extracted from the obtained cake with a solvent. The changes in the quality of both crude oils obtained by pressing and by solvent were investigated during refining. Findings revealed that the *p*‐anisidin value (*p*‐AV) and fatty acid profile did not change significantly, but there were significant differences (*p* < .05) in the peroxide value (PV), reflective index, pigment contents, free fatty acid content (FFA%), and antioxidant activity (total phenol content (TPC), thymoquinone, and DPPH inhibition) of BCS oils obtained by the two different methods. PV and FFA decreased to less than 15 meqO_2_/kg and 0.3%, respectively, in the refined oil. The TPC (65%), thymoquinone (45–97%), carotenoids (86–89%), and chlorophyll (75–85%) were removed from BCS oil, but the DPPH value was raised by about 33%. The current study gives a clear picture of the changes during refining in BCS oil, which can be a useful guide in food applications.

## Graphical abstract

Black cumin seed (BCS) oil has many benefits as it is rich in bioactive compounds. However, its high peroxide value and acidity limit its applications. In this investigation, hot press oil had higher bioactive components than solvent‐extracted oil. So, refining can be offered to improve the BCS oil quality for various applications.

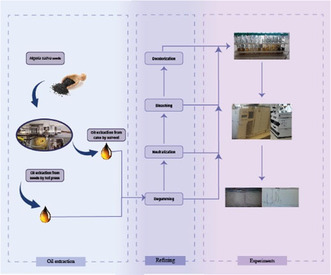

## 1 INTRODUCTION

Black cumin (*Nigella sativa* L.) is an herbaceous plant in the Ranunculaceae family with a long history of medical use, with seeds containing a high concentration of bioactive components (Bakhshabadi et al., 2017). Black cumin seed (BCS) is found in a wide range of recipes as a spice or flavoring component. Furthermore, BCS has a relatively high oil content (25–40%), with various uses and applications in the food, cosmetics, and pharmaceutical sectors (Suri et al., 2019).

Oil from oilseeds can be extracted by pressing or using a solvent. Pressing is employed for seeds with a high oil content (more than 20%); nevertheless, this approach has a relatively low oil extraction yield and leaves a large amount of oil in the press cake, which is then extracted using a solvent such as hexane (Bhuiya et al., 2020).

Cold and hot presses are two techniques used in oil extraction by press. A cold press extracts oil from oilseeds at low temperatures (below 40°C), whereas a hot press extracts oil at high temperatures. In the oil industry, hot pressing, which is more profitable commercially, yields more oil than cold pressing (Wahidu et al., 2014).

Compared with the oil extracted by cold press, both the oil extracted by hot press, due to the heat generated during the processing, and the oil extracted by a solvent, due to the solubility of the components, contain many impurities, such as pigments, free fatty acids, gums, residual pesticides, and metals. For these reasons, it seems necessary to apply the refining process to pressed hot and solvent‐extracted oils to remove impurities and enhance oxidative stability (Santiworakun et al., 2022). Typically, crude oils are refined to remove unwanted compounds with minimal damage to beneficial components and oil loss. Vegetable oil refining often includes degumming, neutralization, bleaching, and deodorization (Chew et al., 2016).

BCS oils extracted by different methods also have weak oxidative stability and high peroxide and acid values; therefore, their uses in various applications are limited. To the best of our knowledge, no information is available regarding the impact of refining procedures on the quality of BCS oil. The objectives of the present study were to examine the impact of various processing stages, including oil extraction by hot press from BCS, oil extraction from the obtained cake from hot press by solvent, and then the refining process, on the quality attributes, antioxidant activity, and bioactive constituents of the extracted oils.

## 2 MATERIALS AND METHODS

### 2.1 Chemicals

The cleaned BCSs were obtained from a local farm in Hamedan (Iran). The activated bleaching earth (Al_2_Si_2_O_5_(OH)_4_) came from the BHD company in Perak, Malaysia. The following items were bought from Sigma‐Aldrich in the USA: chloroform, *n*‐hexane, glacial acetic acid, potassium iodide, ethanol 96%, sodium thiosulfate, starch solution, phenolphthalein indicator, sodium chloride, and the fatty acid methyl ester 37 component mix source material. Throughout the entire study, analytical‐grade compounds were used.

### 2.2 Oil extraction from black cumin seeds

**FIGURE 1:**
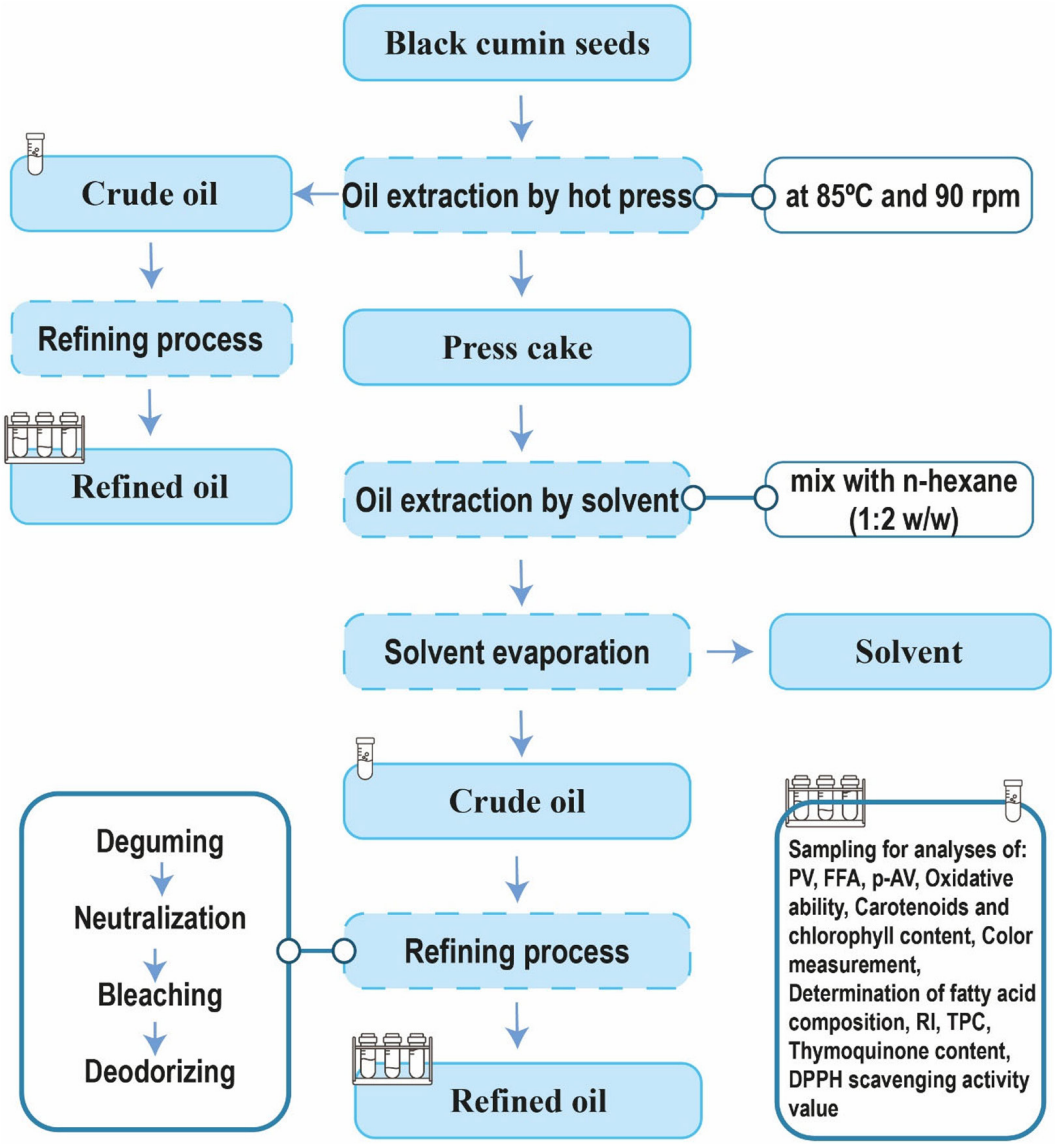
Black cumin seed oil extraction and refining flow chart.

Figure 1 shows the flow chart of the methods applied for oil extraction from BCSs. The cleaned BCSs were hot pressed, and the cake obtained from the press was subjected to oil extraction by solvent. The extracted crude oils (by hot press from the seeds and by a solvent from the cake) were refined, and the obtained oils were analyzed for their quality as explained below for the oil extraction yield, peroxide value (PV), free fatty acid (FFA), *p*‐anisidine value (*p*‐AV), refractive index (RI), oxidation stability, color measurement, carotenoids and chlorophyll content, fatty acid composition, polyphenols, thymoquinone, and DPPH scavenging activity value (Azadmard‐Damirchi & Dutta, 2007).

### 2.3 Hot press of BCSs

The BCSs were pressed by a screw press (Screw Press Model 85 mm, Kern Kraft, Germany) according to the previously published methods at 85°C and 90 rpm (Wahidu et al., 2014). The obtained crude oil was stored at 4°C for quality assessment and the refining process.

### 2.4 Oil extraction by solvent from BCS cake

The residual oil in the cake remaining from hot‐pressed BCSs was extracted by solvent (*n‐*hexane) according to the Pan et al. (2019) method. The cake was ground by a grinder (Asantoos, Iran) and mixed with *n*‐hexane (1:2 w/w). Then, the mixture was shaken for an hour and then filtered; next, the solvent was removed by a rotary evaporator under a vacuum. The extracted oil samples were kept at 4°C until further testing.

### 2.5 The extraction yields

The yield of BCS oil extracted from the two steps described above was determined as follows (Ashrafi et al., 2023):

(1) $$\text{Yield}\;\left(Y\right)=\frac{\text{weight of the extracted oil}}{\text{weight of seeds or cake}\;}\times 100$$

### 2.6 The oil‐refining process

Oil refining was performed on a laboratory scale according to the previously published method (Azadmard‐Damirchi & Dutta, 2007). Phosphoric acid (85%) was added during the degumming process, followed by water washing. NaOH (10% in water) was used for the neutralization of the degummed oil, and then the neutralized oil was washed with water. In the next step, 1% (wt) of the bleaching earth was mixed with the oil to perform bleaching and produce bleached oil. The bleached oils were then deodorized under vacuum at 180°C.

### 2.7 Peroxide value (PV)

The AOCS (2017) official technique (Cd 8b‐90) was used to determine the peroxide value of the oil samples by titration with sodium thiosulfate (0.1 N).

### 2.8 Free fatty acid content

The FFA content was determined by titration using the AOCS (2017) method (Cd 8b‐90).

### 2.9 *p*‐Anisidine value (*p*‐AV)

The anisidine value was measured at 350 nm in a 1.0 cm cell containing 1.0 g of oil dissolved in 100 mL of isooctane using the Cd 18‐90 (AOCS, 2017).

### 2.10 Oxidative stability

The oxidative stability of the oil samples was evaluated using the 743 Professional (Rancimat from Metrohm, Herisau, Switzerland) according to the previously published method (Piravi‐vanak et al., 2022). The temperature of the heating block was set at 110°C, and the airflow rate was set at 10 L/h.

### 2.11 Carotenoids and chlorophyll content

According to the guidelines presented by Minguez‐Mosquera et al. (1991), the Thermo Scientific UV–Visible Spectrophotometer was used to determine the amounts of carotenoids and chlorophyll. 2 mL of cyclohexane was used to completely dissolve a 2 g sample of the oil. The solution absorbance was measured at wavelengths of 470 nm for carotenoids and 670 nm for chlorophylls. The computation of the pigment content was done as follows:

(2) $$\text{Chlorophyll}\;\left(\mathrm{mg}/\mathrm{kg}\;\text{of oil}\right)=\left(A670\times 10^{6}\right)/\left(613\times 100\times d\right)$$

(3) $$\text{Carotenoids}\;\left(\mathrm{mg}/\mathrm{kg}\;\text{of oil}\right)=\left(A470\times 10^{6}\right)/\left(2000\times 100\times d\right)$$

A is the sample absorbance; 613 denotes the extinction coefficient of the chlorophyll; 2000 represents the extinction coefficient of the carotenoids; and *d* denotes the thickness of the spectrophotometer cell (1 cm).

### 2.12 Color measurement

The color estimated by a Lovibond (Lovibond PFX996, Tintometer) of extracted oils and during refining was analyzed following the methods of AOCS (2017) (Cc 13b‐45).

### 2.13 Determination of fatty acid composition

Gas chromatography (Agilent 8790B) combined with an FID detector and BPX70 (60 m × 0.25 mm i.d., 0.25 μm) capillary column was used to evaluate the fatty acid composition of the samples according to the previously published method (Fathi‐Achachlouei et al., 2019). NaOCH_3_ was given an addition of around oil in a methanolic solution and hexane. After sonication and filtering, the supernatant phase of the combination was injected into the GC.

### 2.14 Refractive index (RI)

RI was determined using a refractometer at 20°C by AOCS (2020).

### 2.15 Total polyphenol content (TPC)

The Folin–Ciocalteau colorimetric technique was used to investigate TPC (Bail et al., 2008).

### 2.16 Thymoquinone content

The thymoquinone in the samples was determined using the technique reported by Alkhatib et al. (2020) by HPLC.

### 2.17 DPPH‐scavenging activity value

The radical scavengers activity of the oils was investigated by decreasing DPPH in toluene using the method described by Ramadan and Mörsel (2004).

### 2.18 Statistical evaluation

Each experiment was done in triplicate, and the results were represented as the mean and the standard deviation (SD) of the mean. The statistical analysis of the gathered data was done using GraphPad Prism 9. The one‐way ANOVA statistical analysis was followed by the Duncan test to compare means and evaluate the statistical significance as differences at or above .05.

## 3 RESULTS AND DISCUSSION

### 3.1 Yield of extraction

Several factors influence the vegetable oil extraction yield, including oilseed type, oilseed moisture content, temperature, pressure during the press, and extraction process. The oil extracted by hot pressing from the seeds and by solvent from the press cake was 37% and 8%, respectively. Overall, 45% of the oil was obtained from the *Nigella sativa* L. seeds. Generally, the obtained results are in agreement with the previously published data. There are several researches on oil extraction by press from the BCS that report its oil percentage from 34.4 to 38.7% (Gharby et al., 2015; Mazaheri et al., 2019a; Sakdasri et al., 2023).

### 3.2 Peroxide value

**FIGURE 2:**
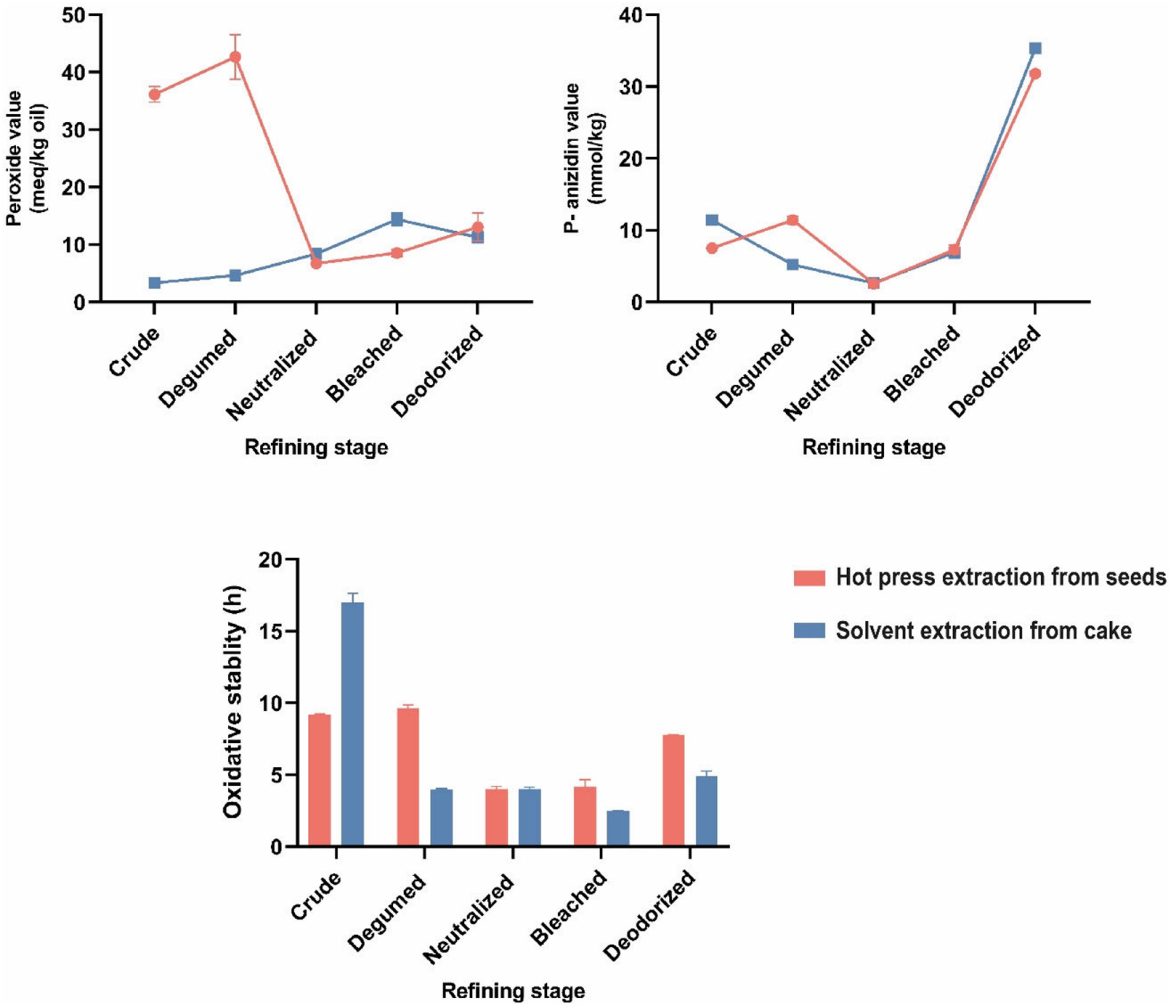
Peroxide, *p*‐anisidine values, and oxidative stability (Rancimat) of black cumin seed oil extracted by hot‐press extraction and solvent extraction of hot‐pressed cake during the refining process.

PV is one of the crucial markers for determining the quality of edible oils. The PV of hot press BCS oil and solvent‐extracted BCS oil from hot‐pressed cake were 36.14 and 3.39 (meq/kg oil), respectively (Figure 2). Therefore, to make the hot press BCS oil usable, it needs to be refined to reduce the PV to an acceptable level. There are many reports showing that BCS oil has high PV after extraction by pressing (Soleimanifar et al., 2019).

In order to control the PV and prevent its increase, several pretreatments were performed before oil extraction from BCS. Microwave and roasting were used to inactivate the enzymes in BCS to extract oil with low PV by pressing (Mazaheri et al., 2019a). However, it has been reported that the PV of BCS oil increased (13.15 meqO_2_/kg oil) when the microwave power and processing time increased during the pretreatment of BCS (Bakhshabadi et al., 2017).

Vegetable oils extracted using a hot press or a solvent are refined to eliminate contaminants and oxidation products (Vaisali et al., 2015). The obtained results showed that the PV of the hot‐press oil decreased from 36 to 13 (meqO_2_/kg oil) after applying the refining process (Figure 2). The neutralizing step had the greatest impact on PV reduction because it removed the hydroperoxide compounds trapped in the oil by soap. According to the reported PV by Sánchez‐Machado et al. (2015), there were notable variations in the PV between the various steps of Moringa oleifera seed oil refining. The PV of the soybean and canola oils was investigated by Farhoosh et al. (2009) after each processing step of refining. The crude oils had a PV of less than 2 (meqO_2_/kg oil), and their levels did not differ significantly after neutralization.

Wu et al. (2019) also showed that bleaching and deodorization greatly reduced the PV of all types of rapeseed oils, while degumming and neutralization significantly raised it. Probably the production of the primary oxidation products brought on by lipoxygenases, air, heat, moisture, and light activities is what causes the increase in the PV of all types of crude rapeseed oils during degumming–neutralization. Moreover, the absorbance and catalysis of peroxide molecules by bleaching earth are responsible for a considerable fall in the PV of bleached oils following neutralization. Deodorization considerably reduced the PV of all types of bleached rapeseed oils, which might be attributable to the peroxides' volatilization and subsequent breakdown.

### 3.3 *p*‐Anisidine value

The *p*‐AV measures the secondary oxidation products, which are formed by the degradation of peroxides in oils. The *p*‐AV of the hot‐pressed BCS oil and the solvent‐extracted BCS oil from the cake was 7.51 and 11.4 mmol/kg, respectively. These results show that some hydroperoxides in the hot‐pressed cake were converted to aldehydes. The *p*‐AV of BCS oil was reported to be less than 50 (mmol/kg), which is consistent with current research findings (Ramadan & Mörsel, 2004).

The absorption of aldehydes in soap and gum during the degumming and neutralization stages of refining lowered *p*‐AV in the oil. On the other hand, during bleaching and deodorizing, *p*‐AV rose when hydroperoxides were changed into aldehydes. The *p*‐AV was significantly (*p* < .05) decreased throughout the chemical refining of kenaf seed oil from crude oil to degummed oil, while neutralization and bleaching significantly (*p* < .05) enhanced the oil's *p*‐AV. It was observed that it might be related to the catalytic property of acid‐activated bleaching earth, which converts hydroperoxides into secondary oxidation products, including aldehydes and ketones (Chew et al., 2016). An additional investigation found that the processes of acid degumming and neutralization with 15°Bé NaOH and bleaching with acid‐activated bleaching earth had successfully produced edible *Moringa oleifera* kernel oil with suitable physicochemical and oxidative qualities. In comparison to deodorization, neutralization had a stronger impact on lowering the oil acid value and *p*‐AV. This, together with the high levels of tocopherols (65–87 mg/kg) and oleic acid (80%), led to the oil's high level of oxidative stability (Abd Hadi et al., 2021).

### 3.4 Oxidation stability

Oxidative stability is the time period that is assessed by Rancimat. During that time, one oxidation factor, including the peroxide value or carbonyl quantity, rapidly rises and gives the oil an unfavorable flavor and odor. The profile of fatty acids, the presence of antioxidants, and other minor components (e.g. iron and cupper ions) are the key factors influencing the oxidative stability of vegetable oils. According to the obtained results, the oxidative stability of the hot‐pressed BCS oil was lower (9 h) than that of the solvent‐extracted oil from the cake (17 h). In another study, using various pretreatments such as microwave and pulsed electric fields increased the oxidative stability of BCS oil (Bakhshabadi et al., 2018). However, after the use of refining techniques, this ratio was reversed and the hot‐pressed oil was more stable than the solvent‐extracted BCS oil from the cake (Figure 2). There was no strong correlation found between oxidative stability and the significant classes of TPC and thymoquinone, which is in accordance with the results of Ayyildiz et al. (2015), while the reduction of peroxide and free fatty acids is in line with the high oxidative stability. The reduction in the concentration of antioxidant compounds after applying the refining process is the main reason for reducing the oxidative stability of refined oils compared to crude oils.

### 3.5 The free fatty acid (FFA) content

One of the other major quality indices of vegetable oils is FFA%, which demonstrates the hydrolysis reaction of triacylglycerol structure and is calculated as a percentage of the dominant fatty acid in a specific oil. The FFA% of BCS oil obtained from the two different extractions was 2% and 7% for the hot press and solvent extraction from the cake, respectively (Table 1). These results are comparable with the previous research, which reported the level of FFA% for cold‐pressed, solvent‐extracted, and microwave‐assisted BCS oil as 7.5%, 9.5%, and 9%, respectively (Kiralan et al., 2014). Following the neutralization, the FFA% in BCS crude oil was neutralized by sodium hydroxide and was decreased to 0.2%–0.25%. (Table 1). After the bleaching step, the FFA% of BCS oil increased to 0.25%, and 0.4% was theoretically achievable due to the ester hydrolysis carried out by the bleaching acidified earth.

**TABLE 1:** Physicochemical properties of BCS oil extracted by hot‐press extraction and solvent extraction of hot‐pressed cake during the refining process.

	Free fatty acids content (%)		Reflective index		Colorimetry					
					l*		a*		b*	
	HPE	SEHPM	HPE	SEHPM	HPE	SEHPM	HPE	SEHPM	HPE	SEHPM
Crude	2.04 ± 0.02^aB^	6.74 ± 0.09^aA^	1.471 ± 0.0001^aA^	1.461 ± 0.0001^eB^	6.69 ± 0.15^cdB^	88.46 ± 1.20^aA^	3.34 ± 0.07^bA^	−4.53 ± 0.16^bcB^	4.68 ± 0.10^cB^	9.91 ± 0.30^aA^
Degummed	1.8 ± 0.24^bB^	6.54 ± 0.01^aA^	1.4715 ± 0.0001^aA^	1.463 ± 0.0005^dB^	1.80 ± 0.00^dB^	83.27 ± 6.80^aA^	1.08 ± 0.04^dA^	−5.29 ± 0.56^cdB^	1.18 ± 0.05^dB^	8.10 ± 0.88^aA^
Neutralized	0.18 ± 0.01^cA^	0.25 ± 0.05^bA^	1.471 ± 0.0001^aA^	1.464 ± 0.0001^cB^	12.01 ± 1.18^cB^	71.42 ± 0.86^bA^	7.57 ± 0.63^aA^	−5.91 ± 0.14^dB^	7.93 ± 0.88^bcA^	6.95 ± 0.19^aA^
Blenched	0.24 ± 0.03^cA^	0.38 ± 0.03^bA^	1.471 ± 0.0001^aA^	1.4703 ± 0.0006^bB^	34.28 ± 0.15^bB^	62.08 ± 4.19^cA^	3.26 ± 0.34^bA^	−4.04 ± 0.29^bB^	11.08 ± 0.17^bA^	4.86 ± 0.54^bB^
Deodorized	0.22 ± 0.04^cA^	0.35 ± 0.02^bA^	1.471 ± 0.0001^aA^	1.471 ± 0.0001^aA^	48.31 ± 6^aB^	61.57 ± 0.71^cA^	8.24 ± 1.22^aA^	3.01 ± 0.06^aB^	32.40 ± 4.01^aA^	5.1 ± 0.13^bB^

*Note*: Means ± standard deviations followed by different superscript lowercase letters (a–e) within the same column and capital letter within each row (A–B) are significantly different according to Tukey's test (*p* < .05).

Abbreviations: HPE, Hot press extraction; SEHPM, Solvent extraction of hot‐pressed cake.

The same changing procedure was observed during the refining of evening primrose oil. The acid value (AV) of evening primrose crude oil was 1.88 mg KOH/g. As a result of sodium hydroxide neutralizing of the FFAs during the deacidification stage of the refining process, the acid value dropped to 0.19 mg KOH/g. The ester hydrolysis caused by the bleaching earth during the refining resulted in a small rise in the AV of the oil to 0.45 mg KOH/g. On the other hand, it can also be due to the adsorbent that was employed. The generation of FFAs and a rise in the AV were caused by the adsorption of sodium ions in soap during the decolorization process. The fully refined oil AV was only 0.37 mg KOH/g, which was lower than expected, which indicates an improvement in the oil quality (Pan et al., 2020).

### 3.6 Carotenoids and chlorophyll content

Carotenoids are useful bioactive compounds in vegetable oils that act as antioxidants and enhance the oxidative stability of vegetable oils (Blasi & Cossignani, 2020). Chlorophylls can, however, cause sensitization and start the photooxidation of vegetable oils. As a result, reducing chlorophyll can improve the shelf life of vegetable oils (Rukmini & Raharjo, 2010).

**FIGURE 3:**
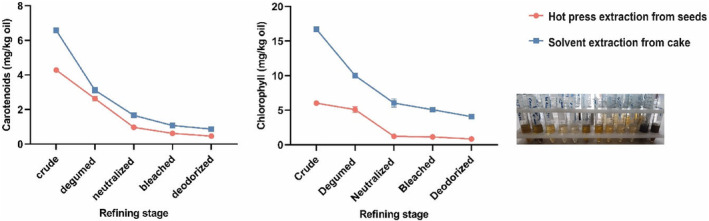
Chlorophyll and carotenoid contents of BCS oil extracted by hot press extraction and solvent extraction of hot‐pressed cake during the refining process.

Carotenoids and chlorophyll were identified at the highest concentrations in the solvent‐extracted BCS oil, at approximately 6.57 and 16.72 mg/kg, respectively. At the same time, lower amounts were noticed in the hot‐pressed BCS oil, at 4.28 and 6.02 mg/kg, respectively (Figure 3). As these pigments are soluble in n‐hexane, their content is higher in the solvent‐extracted black cumin seed oil.

The chlorophyll content of the hot‐pressed BCS oil is similar to the amount reported by Cheikh‐Rouhou et al. (2007), but it is higher than the chlorophyll content of the BCS oil extracted by various methods reported by Kiralan et al. (2014). This variation is most likely related to the variety of the seeds and the differences in the extraction conditions.

Several studies found that the refining process, especially bleaching, had a significant impact on the oil pigments, including chlorophyll and carotenoids. In the solvent‐extracted BCS oil, the chlorophyll and carotenoid levels were significantly reduced in the degumming step. At the same time, neutralization was an efficient procedure for the reduction of hot‐pressed BCS oil pigments. In both types of the extracted BCS oils, the lowest concentration of these compounds was determined during the deodorization step. In another study, however, the chlorophyll and carotenoids in sunflower and rapeseed oils were significantly removed during the bleaching stage of the refining process (Kreps et al., 2014). The impact of refining on the amount of carotenoids in palm oil was studied by Szydłowska‐Czerniak et al. (2011). The results showed that the refining procedure reduced the quantity of carotenoids by over 99% for the investigated palm oils.

### 3.7 Color measurement

The values of the Hunter colors were given as L* (lightness to darkness), greenness (−a*) to redness (+a*), and yellowness (−b*) to blueness (+b*). L* (6.7, 88.5), a* (3.3, −4.5), and b* (4.7, 9.9), respectively, were the color values of the crude oils obtained by hot pressing the seeds and solvent extracting the cake (Table 1). According to the above data, the BCS oil extracted from the cake using a solvent was darker, greener, and yellower than the BCS oil produced using the hot press. Further data from the color characteristics of crude BCS oil, which concern unroasted seeds and are more similar to the hot‐pressed BCS oil demonstrated in this study, have been reported (Suri et al., 2019). The color of vegetable oils can be affected by their pigment types and their content. Carotenoids and chlorophyll are the two main pigments in BCS oil, which affect the color of this oil. Based on the findings, these two compounds were reduced during the refining procedure, which also explains why the color of the refined BCS oil was changed. Chew et al. (2017) determined that color loss was enhanced and increased with bleaching earth concentrations. This is due to the increased surface area for pigment adsorption, resulting in more color reduction.

### 3.8 Fatty acid composition analysis

The fatty acid composition of vegetable oils can have a direct effect on the quality of the oils and fats, as well as a significant impact on the oil oxidation stability. Analysis of the results obtained from the gas chromatography showed that linoleic acid (C18:2) and oleic acid (C18:1) were the two main unsaturated fatty acids in the BCS oil, which were detected at about 52% and 26%, respectively (Table 2). Palmitic acid (C16:0) was the most common saturated fatty acid (14%). Results also showed that there were no significant differences (*p* < .05) in the fatty acid composition of the oil extracted by press or solvent.

**TABLE 2:** Fatty acid composition of hot‐press BCS oil extracted and solvent extraction of hot‐pressed cake during the refining process.

	Myristic acid (14:0)		Palmitic acid (16:0)		Palmitoleic acid (16:1)		Stearic acid (18:0)		Oleic acid (18:1)		Linoleic acid (18:2)		Linolenic acid (18:3)		Arachidic acid (20:0)		Gadoleic acid (20:1)		Behenic acid (22:0)	
	HPE	SEHPM	HPE	SEHPM	HPE	SEHPM	HPE	SEHPM	HPE	SEHPM	HPE	SEHPM	HPE	SEHPM	HPE	SEHPM	HPE	SEHPM	HPE	SEHPM
Crude	0.15 ± 0.01^bB^	0.25 ± 0.02^bA^	14.58 ± 0.02^aA^	13.23 ± 0^dB^	0.24 ± 0.008^aA^	0.22 ± 0.005^bcB^	3.78 ± 0.05^aA^	3.63 ± 0.02^aB^	25.48 ± 0.01^aB^	26.61 ± 0.08^aA^	52.94 ± 0.15^dA^	52.38 ± 0.06^bB^	0.183 ± 0.007^dB^	0.23 ± 0.01^bA^	0.13 ± 0.032^cA^	0.13 ± 0.022^aA^	0.24 ± 0.032^dB^	0.31 ± 0.03^aA^	2.35 ± 0.08^bB^	2.84 ± 0.16^aA^
Degummed	0.21 ± 0.03^aA^	0.24 ± 0.01^bA^	14.32 ± 0^aB^	17.4 ± 0.09^aA^	0.23 ± 0.004^abA^	0.20 ± 00.007^cdB^	3.85 ± 0.05^aA^	3.14 ± 0.1^cB^	25.52 ± 0.04^aA^	25.11 ± 0.1^bB^	52.77 ± 0.07^dA^	51.50 ± 0.07^dB^	0.25 ± 0.02^bA^	0.16 ± 0.01^cB^	0.18 ± 0.015^bA^	0.17 ± 0.032^aA^	0.27 ± 0.015^cdA^	0.22 ± 0.045^bB^	2.37 ± 0.14^abA^	1.83 ± 0.13^cB^
Neutralized	0.18 ± 0.02^abA^	0.19 ± 0^cA^	12.96 ± 0.04^bB^	14.04 ± 0.09^cA^	0.21 ± 0.009^bcA^	0.19 ± 0.002^dB^	3.74 ± 0.1^aA^	3.46 ± 0.05^bB^	25.29 ± 0.05^aB^	26.52 ± 0.05^aA^	53.78 ± 0.11^bA^	52.08 ± 0.06^dB^	0.52 ± 0.02^aA^	0.21 ± 0.01^bB^	0.21 ± 0.02^bA^	0.14 ± 0.015^aB^	0.41 ± 0.02^aA^	0.29 ± 0.015^aB^	2.67 ± 0.16^abA^	2.78 ± 0.07^aA^
Blenched	0.13 ± 0^bcA^	0.16 ± 0.02^cA^	12.88 ± 0.03^bA^	12.83 ± 0.16^eA^	0.21 ± 0.003^bcB^	0.229 ± 0.007^bA^	3.58 ± 0.03^bB^	3.62 ± 0.04^aB^	24.83 ± 0.06^bB^	26.6 ± 0.07^aA^	54.80 ± 0.07^aA^	52.92 ± 0.07^aB^	0.22 ± 0.01^cA^	0.22 ± 0.015^bA^	0.39 ± 0.01^aA^	0.16 ± 0.02^aB^	0.31 ± 0.024^bcA^	0.27 ± 0.02^abB^	2.59 ± 0.09^abB^	2.98 ± 0.22^aA^
Deodorized	0.10 ± 0.01^cB^	0.34 ± 0.04^aA^	13.08 ± 0.06^bB^	16.43 ± 0.10^bA^	0.2 ± 0.005^cB^	0.3 ± 0.008^aA^	3.87 ± 0.03^aA^	3.44 ± 0.06^bB^	25.67 ± 0.06^aA^	24.61 ± 0.07^cB^	53.55 ± 0.08^cA^	51.63 ± 0.1^dB^	0.25 ± 0.01^bcB^	0.3 ± 0.01^aA^	0.17 ± 0.03^bA^	0.17 ± 0.01^aA^	0.34 ± 0.03^bA^	0.32 ± 0.01^aA^	2.71 ± 0.2^aA^	2.4 ± 0.13^bB^

*Note*: Means ± standard deviations followed by different superscript lowercase letters (a–e) within the same column and capital letter within each row (A–B) are significantly different according to Tukey's test (*p* < .05).

Abbreviations: HPE, Hot press extraction; SEHPM, Solvent extraction of hot‐pressed cake.

The results show that the primary fatty acid composition of BCS oil, including linoleic, oleic, and palmitic acids, remained unchanged throughout the refining procedure (Table 2). These results agree with the findings of the refining effects on the sunflower, hazelnut, and evening primrose oil fatty acid compositions (Durmaz & Gökmen, 2019; Pal et al., 2015; Pan et al., 2020).

### 3.9 Refractive index

The RI value for the hot‐press BCS oil (1.471) was higher than that of the solvent‐extracted oil from BCS cake (1.46), which is consistent with the values reported by Kiralan et al. (2014). The RI barely changed while the solvent‐extracted BCS oil from the cake was being refined. The RI can be affected mainly by the fatty acid composition. The results obtained for RI are also supported by the results of the fatty acid composition since there were no appreciable changes in the fatty acid composition throughout refining (Sánchez‐Machado et al., 2015).

### 3.10 Total phenol content (TPC) analysis

**FIGURE 4:**
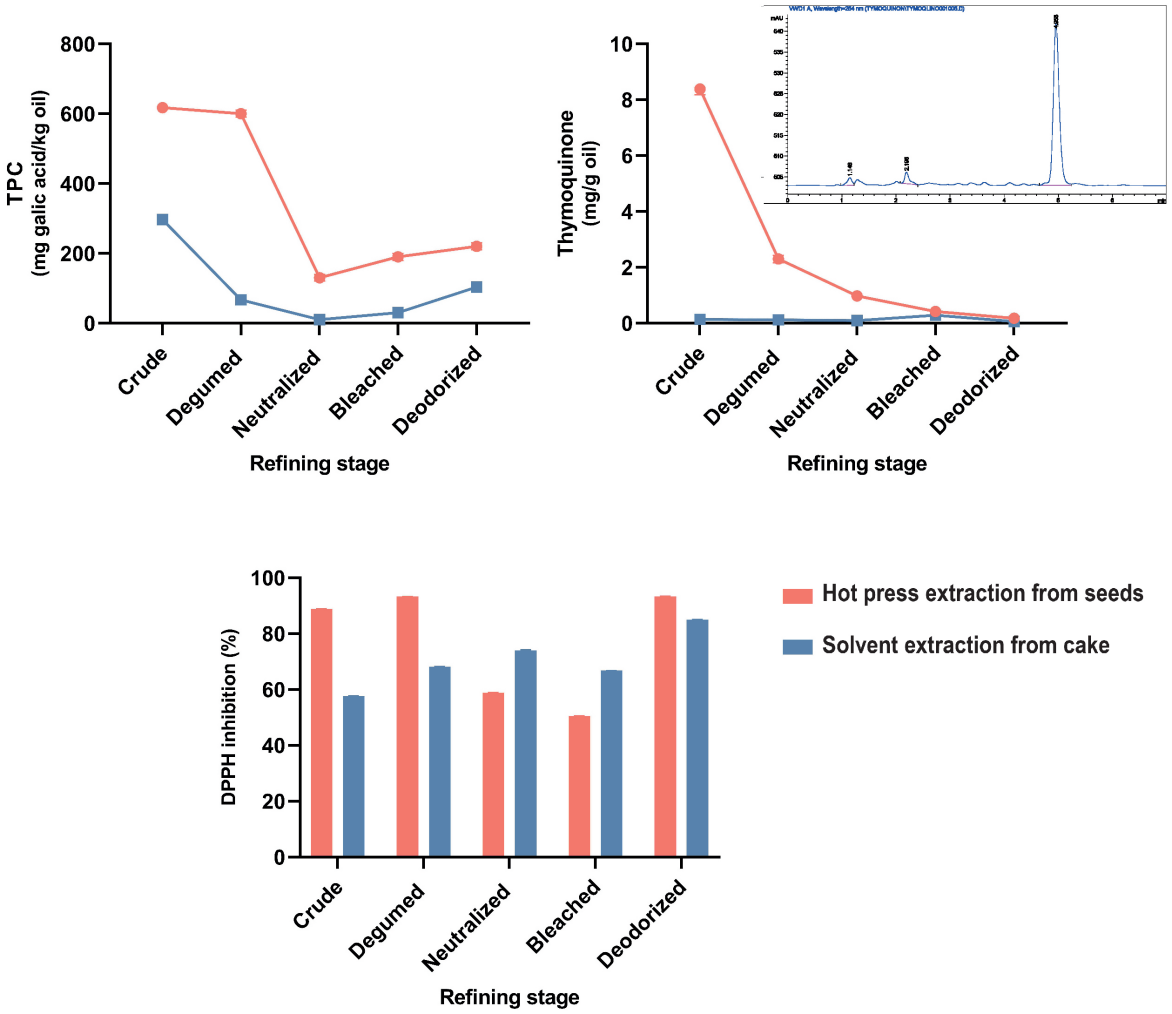
TPC (total phenol content), thymoquinone, and DPPH (2,2‐diphenyl‐1‐picrylhydrazyl) inhibition of BCS oil extracted by hot‐press extraction and solvent extraction of hot‐pressed cake during the refining process.

TPC is a significant quality criterion that reveals the ability of an oil to remain fresh for a long time, as well as its organoleptic and nutritional value. According to the TPC analysis of the BCS oils produced by hot pressing and solvent extraction of the cake (Figure 4), the concentration of total phenols in the hot‐pressed BCS oil was much greater than that of the solvent‐extracted BCS oil. Moreover, it surpassed the TPC content of the cold‐pressed BCS oil (598.4 mg caffeic acid/kg oil) published by Mazaheri et al. (2019b). In another study, He et al. (2022) reported that hot‐pressed rapeseed oil contains more polyphenols than cold‐pressed oil.

Generally, degumming, neutralization, and bleaching had profound effects on the TPC, and 65% of the TPC was lost during these steps (Figure 4). Deodorization did not decrease the TPC of the BCS oil (Figure 4). The same results were observed by Zacchi and Eggers (2008) during rapeseed oil refining. Neutralization of rice bran oil resulted in the most significant decrease in polyphenols. BCS oil contains phenols that are highly soluble in water, which can cause water degumming to lower TPC. This is because polyphenols are polar molecules and weak acids, making it easy to extract them from oils using aqueous solutions, especially when neutralized by sodium hydroxide (Liu et al., 2019). Also, the absorption of the bleaching earth can reduce the content of total phenols.

### 3.11 Thymoquinone content

Thymoquinone is the compound responsible for the biological activity and most health advantages of black cumin seed and its oil. The thymoquinone concentration of hot‐pressed BCS oil and solvent‐extracted oil from the cake was determined in the current investigation. The maximum concentration of thymoquinone was detected in the hot‐pressed crude oil (8.32 mg/g), which was comparable to the work of Lutterodt et al. (2010). The thymoquinone concentration of the solvent‐extracted BCS oil from the cake was 0.14 mg/g oil (Figure 4). This average concentration was lower than the 1.06 mg/g reported by Solati et al. (2014) in the BCS oil obtained by Soxhlet.

The changes in the thymoquinone content were determined in the BCS crude oil and at each stage of the refining process (Figure 4). Particularly, the refining process caused an almost 97.9% decrease of thymoquinone in the hot‐press BCS oil and a 57.14% decrease in solvent‐extracted BCS oil from the cake. This might be related to the instability of thymoquinone during the oil refining process. The most significant thymoquinone losses were found during the degumming process of the hot‐pressed BCS oil, followed by the neutralization process. There is no comparison of changes in the thymoquinone content during refining with those of the earlier publications due to the lack of previous investigations on the influence of refining on BCS oil.

### 3.12 DPPH‐scavenging activity value

The DPPH radical test is one of the most often used methods for expressing antioxidant activity and comparing the antioxidant capacity of different materials (Locatelli et al., 2009).

In confirmation of the results of TPC and thymoquinone contents, it has been found that inhibition of DPPH free radicals in the BCS oil extracted by hot pressing was better than that in the oil extracted from the cake by solvent (Figure 4). The antioxidant activity of the hot‐pressed BCS oil was the same as the six cold‐pressed BCS oils studied in another study by Lutterodt et al. (2010).

DPPH techniques were used to assess the antioxidant properties of the BCS oils at various phases of the refining process using these different extraction methods, and the findings were compared (Figure 4). After the neutralization and bleaching stages, the oils obtained from BCS showed the highest loss in antioxidant activity. In contrast, an increase in DPPH free radical inhibition capability was observed following the deodorization step. Most of the investigations showed a reduction in edible oils' antioxidant activity during refining due to the loss of bioactive compounds (Durmaz & Gökmen, 2019; Pan et al., 2020). However, some studies have found that antioxidant activity increases during some stages of refining. For example, Zarei Jelyani et al. (2021) reported an increase in the bleaching stage of Baneh kernel oil refining, and an increase was observed in kenaf oil by Chew et al. (2016).

## 4 CONCLUSIONS

BCS has a high content of oil with many bioactive compounds, which makes it very useful to be used in different applications. In this study, BCS oil was extracted by the hot press, which gave 37% oil. Also, the obtained cake was extracted by a solvent, which yielded 8% oil. The hot‐pressed crude oil had higher PV, RI, TPC, thymoquinone, and DPPH inhibition, and lower *p*‐AV, FFA%, oxidative stability, carotenoids, and chlorophyll than the solvent‐extracted oil. The obtained oils were refined, and the results showed that the PV, FFA, TPC, thymoquinone, carotenoids, and chlorophyll were reduced to 55%, 95%, 65%, 45–97%, 86–89%, and 75–85%, respectively. Finally, it can be concluded that the oil extracted from BCS has a high PV and *p*‐AV. Therefore, refining has to be done to reduce the PV and FFA%, which can expand this oil application.

## AUTHOR CONTRIBUTIONS

**Solmaz Abedinzadeh:** Formal analysis (equal); investigation (equal); writing – original draft (equal). **Mohammadali Torbati:** Investigation (equal); methodology (equal); supervision (equal). **Sodeif Azadmard‐Damirchi:** Conceptualization (equal); investigation (equal); methodology (equal); supervision (equal). **Fataneh Hashempour‐Baltork:** Methodology (equal); writing – review and editing (equal).

## FUNDING INFORMATION

The authors appreciate the support of Tabriz University of Medical Sciences to accomplish this research, grant number 39135.

## CONFLICT OF INTEREST STATEMENT

The authors declare that they have no conflicts of interest in this work.

## Data Availability

Data are contained within the article.
